# Improved conversion efficiency of Ag_2_S quantum dot-sensitized solar cells based on TiO_2 _nanotubes with a ZnO recombination barrier layer

**DOI:** 10.1186/1556-276X-6-462

**Published:** 2011-07-21

**Authors:** Chong Chen, Yi Xie, Ghafar Ali, Seung Hwa Yoo, Sung Oh Cho

**Affiliations:** 1 Department of Nuclear and Quantum Engineering, Korea Advanced Institute of Science and Technology (KAIST), 373-1 Guseong, Yuseong, Daejeon 305-701, Republic of Korea; 2 Nanomaterials Research Group, Physics Division, PINSTECH, Islamabad, Pakistan

**Keywords:** quantum dots, TiO_2 _nanotube, Ag_2_S, solar cells

## Abstract

We improve the conversion efficiency of Ag_2_S quantum dot (QD)-sensitized TiO_2 _nanotube-array electrodes by chemically depositing ZnO recombination barrier layer on plain TiO_2 _nanotube-array electrodes. The optical properties, structural properties, compositional analysis, and photoelectrochemistry properties of prepared electrodes have been investigated. It is found that for the prepared electrodes, with increasing the cycles of Ag_2_S deposition, the photocurrent density and the conversion efficiency increase. In addition, as compared to the Ag_2_S QD-sensitized TiO_2 _nanotube-array electrode without the ZnO layers, the conversion efficiency of the electrode with the ZnO layers increases significantly due to the formation of efficient recombination layer between the TiO_2 _nanotube array and electrolyte.

## Introduction

In recent years, dye-sensitized solar cells (DSSCs) have attracted much attention as a promising alternative to conventional p-n junction photovoltaic devices because of their low cost and ease of production [1-4]. A high power conversion efficiency of 11.3% was achieved [5]. The conventional DSSCs consist of dye-sensitized nanocrystalline TiO_2 _film as working electrode, electrolyte, and opposite electrode. In DSSCs, the organic dyes act as light absorbers and usually have a strong absorption band in the visible. Various organic dyes such as N719 and black dye have been applied for improving the efficiency, light absorption coverage, stability, and reducing the cost. However, the organic dyes have a weak absorbance at shorter wavelengths. Materials that have high absorption coefficients over the whole spectral region from NIR to UV are needed for high power conversion efficiency. During the last few years, instead of organic dyes, the narrow band gap semiconductor quantum dots (QDs) such as CdS [6,7], CdSe [7-9], PbS [10,11], InAs [12], and InP [13] have been used as sensitizers. The unique characteristics of QDs over the organic dyes are their stronger photoresponse in the visible region, tunable optical properties, and band gaps simply by controlling the sizes. The QD-sensitized solar cells (QDSSCs) have been considered the next-generation sensitizers [14]. In either DSSCs or QDSSCs, the nanoparticle porous film electrode plays a key role in the improvement of power conversion efficiency. Recently, to improve the properties of TiO_2 _film electrode, one-dimensional nanostructure arrays as working electrodes, including nanowires and nanotubes, have been proposed and studied. Compared with the nanoparticle porous films, aligned one-dimensional nanostructure arrays can provide a direct pathway for charge transport and superior optical absorption properties. Therefore, more and more studies focus on QDSSCs based on one-dimensional nanomaterials, such as the TiO_2 _nanotubes (TNTs) [15-17].

Among QDs, Ag_2_S is an important material for photocatalysis [18-20] and electronic devices [21-24]. Ag_2_S has a large absorption coefficient and a direct band gap of 0.9 to 1.05 eV, which makes Ag_2_S an effective semiconductor material for photovoltaic application. In the past several years, although there are some reports on the photovoltaic application of Ag_2_S [10,25], few studies on Ag_2_S QDSSCs based on TNTs are reported. In this work, we report on the synthesis of Ag_2_S QD-sensitized TNT photoelectrode combining the excellent charge transport property of the TNTs and absorption property of Ag_2_S. Besides, to improve the efficiency of as-prepared photoelectrodes, we interpose a ZnO recombination barrier layer between TNTs and Ag_2_S QDs to reduce the charge recombination in Ag_2_S QDSSCs because the ZnO layer can block the recombination of photoinjected electrons with redox ions from the electrolyte. Recently, we have reported the improved conversion efficiency of CdS QD-sensitized TiO_2 _nanotube array using ZnO energy barrier layer [26]. Similar method has been used by Lee et al. to enhance the efficiency of CdSe QDSSCs by interposing a ZnO layer between CdSe QDs and TNT [27]. Our results show that Ag_2_S QD-sensitized TiO_2 _nanotube-array photoelectrodes were successfully achieved. The more important thing is that the conversion efficiency of the Ag_2_S-sensitized TNTs is significantly enhanced due to the formation of ZnO on the TNTs.

## Experimental section

### Materials

Titanium foil (99.6% purity, 0.1 mm thick) was purchased from Goodfellow (Huntingdon, England). Silver nitrate (AgNO_3_, 99.5%) and glycerol were from Junsei Chemical Co. (Tokyo, Japan). Ammonium fluoride (NH_4_F), sodium sulfide nonahydrate (Na_2_S, 98.0%), and zinc chloride (ZnCl_2_, 99.995+%) were available from Sigma-Aldrich (St. Louis, MO, USA).

### Synthesis of TNTs

Vertically oriented TNTs were fabricated by anodic oxidation of Ti foil, which is similar to that described by Paulose et al. [28]. Briefly, the Ti foils were first treated with acetone, isopropanol, methanol, and ethanol, followed by distilled (DI) water and finally drying in a N_2 _stream. Then, the dried Ti foils were immersed in high-purity glycerol (90.0 wt.%) solution with 0.5 wt.% of NH_4_F and 9.5 wt.% DI water and anodic oxidized at 60 V in a two-electrode configuration with a cathode of flag-shaped platinum (Pt) foil at 20°C for 25 h. After oxidation, the samples were washed in DI water to remove precipitation atop the nanotube film and dried in a N_2 _stream. The obtained titania nanotube film was annealed at 450°C in an air environment for 2 h.

### Synthesis of Ag_2_S-sensitized plain TNT and ZnO/TNT electrodes

**Figure 1:**
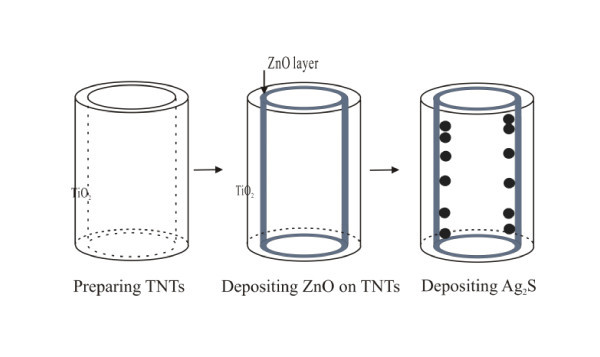
**Preparation process of Ag_2_S quantum dot-sensitized ZnO/TNTs**.

The ZnO thin films on TNTs were prepared by using the successive ionic layer adsorption and reaction method, as described elsewhere [27,29]. Briefly, the annealed TNT electrodes were immersed in 0.01 M ZnCl_2 _solution complexed with an ammonia solution for 15 s and then in DI water at 92°C for 30 s, with the formation of solid ZnO layer. Finally, the as-prepared TNT electrodes were dried in air and annealed at 450°C for 30 min in air for better electrical continuity. Ag_2_S QDs were assembled on the crystallized TNT and ZnO/TNT electrodes by sequential chemical bath deposition (CBD) [25,30]. Typically, one CBD process was performed by dipping the plain TNT and ZnO/TNT electrodes in a 0.1 M AgNO_3 _ethanol solution at 25°C for 2 min, rinsing it with ethanol, and then dipped in a 0.1 M Na_2_S methanol solution for 2 min, followed by rinsing it again with methanol. The two-step dipping procedure is considered one CBD cycle. After several cycles, the sample became dark. In this study, 2, 4, and 8 cycles of Ag_2_S deposition were performed (denoted as Ag_2_S(2), Ag_2_S(4), and Ag_2_S(8), respectively). Finally, the as-prepared samples were dried in a N_2 _stream. The preparation process of as Ag_2_S-sensitized ZnO/TNT electrode is shown in Figure 1. For comparison, Ag_2_S-sensitized TNT electrodes without ZnO films were also fabricated by the same process.

### Materials characterization

The surface morphology of the as-prepared electrodes was monitored using a scanning electron microscope (SEM) (Nova230, FEI Company, Eindhoven, Netherland). The mapping and crystal distribution of the samples were done using a scanning transmission electron microscope (TEM) (Tecnai G2 F30, FEI Company Eindhoven, Netherland) to which an Oxford Instruments (Abingdon, Oxfordshire, UK) energy dispersive X-ray spectroscopy (EDS) detector was coupled. The surface compositions of the samples were analyzed using EDS. The crystalline phase and structure were confirmed by using X-ray diffraction (XRD) (Rigaku D/MAX 2500 V diffractor; Rigaku Corporation, Tokyo, Japan). The UV-visible (UV-vis) absorbance spectroscopy was obtained from a S-4100 spectrometer with a SA-13.1 diffuse reflector (Scinco Co., Ltd, Seoul, South Korea).

### Photoelectrochemical measurements

The photoelectrochemical measurements were performed in a 300-mL rectangular quartz cell using a three-electrode configuration with a Pt foil counter electrode and a saturated SCE reference electrode, and the electrolyte was 1.0 M Na_2_S. The working electrode, including the TNTs, ZnO/TNTs, Ag_2_S(*n*)/TNTs, and Ag_2_S(*n*)/ZnO/TNTs (*n *= 2, 4, and 8), with a surface area of 0.5 cm^2 ^was illuminated under UV-vis light (*I *= 100 mW cm^-2^) with a simulated solar light during a voltage sweep from -1.4 to 0 V. The simulated solar light was produced by a solar simulator equipped with a 150-W Xe lamp. The light intensity was measured with a digital power meter.

## Results and discussion

### Morphology of the TNTs

**Figure 2:**
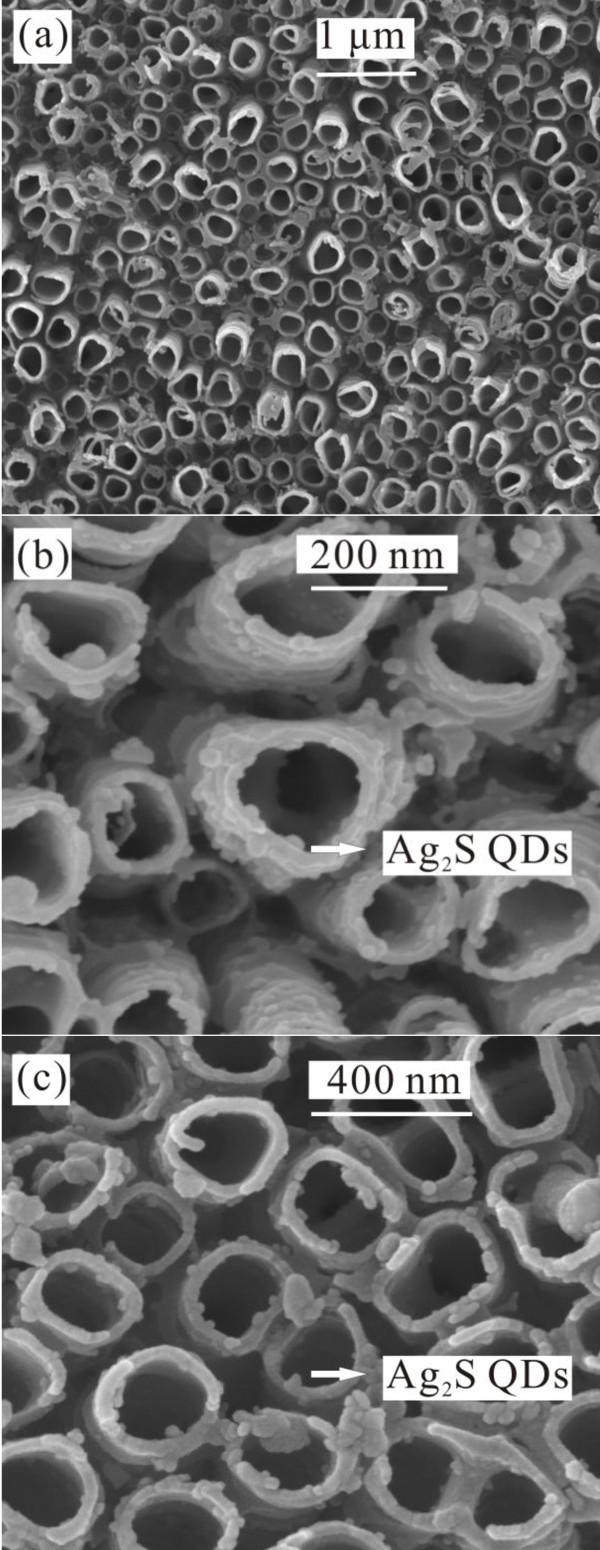
**SEM images of (a) the plain TNTs, (b) Ag_2_S(4)/TNTs, and (c) Ag_2_S(4)/ZnO/TNTs**.

Figure 2a shows the SEM image of the plain TNT film fabricated by anodization of Ti foil before coating with ZnO and Ag_2_S, which reveals a regularly arranged pore structure of the film. The average diameter of these pores is found to be approximately 200 nm and the thickness of the wall of the TNTs approximately 30 nm.

### Characterization of the Ag_2_S QD-sensitized ZnO/TNT (and TNTs) electrodes

Figure 2a shows the surface SEM image of the Ag_2_S(4)/TNT film. It can be clearly seen from Figure 2b that Ag_2_S is deposited as spherical nanoparticles on the TNTs and the wall thickness of the Ag_2_S(4)/TNTs is similar to that of the plain TNTs. In addition, a uniform distribution of the Ag_2_S nanoparticles with diameters of approximately 10 nm is also observed.

For a comparison, the surface SEM image of the ZnO/TNTs covered by Ag_2_S after four CBD cycles (i.e., the Ag_2_S/ZnO/TNT electrode) is shown in Figure 2c. It is found that after the formation of the ZnO thin layer on the TNTs, the diameter and distribution of Ag_2_S nanoparticles did not change much. However, the diameter of the ZnO-coated TNTs increased slightly compared to that of the plain TNTs shown in Figure 2b. These results are similar to previous reports [26,27].

**Figure 3:**
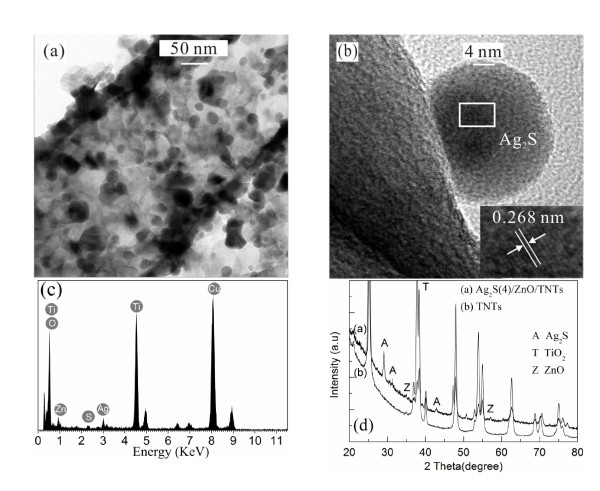
**The low- and high-magnification TEM images, EDX spectrum, and XRD pattern**. (**a**) TEM image of the Ag_2_S(4)/ZnO/TNT electrode showing the formation of ZnO on the TNTs and the Ag_2_S nanoparticles inside the TNTs, (**b**) an HR-TEM image of a deposited Ag_2_S quantum dot, (**c**) the EDX spectrum, and (**d**) XRD pattern of the Ag_2_S(4)/ZnO/TNTs.

The detailed microscopic structure of the Ag_2_S(4)/ZnO/TNTs was further investigated by a high-resolution transmission electron microscope (HR-TEM). Figure 3a shows the low-magnification TEM image of the Ag_2_S(4)/ZnO/TNTs. It can be clearly seen that many Ag_2_S nanoparticles with diameters of approximately 10 nm were deposited into the TNTs. This is supported by our earlier observation in the SEM measurement (Figure 2c). Figure 3b shows the high-magnification image of the Ag_2_S(4)/ZnO/TNTs. It is observed that the crystalline Ag_2_S nanoparticles were grown on crystalline TNTs. In addition, the HR-TEM image in Figure 3b reveals clear lattice fringes, the observed lattice fringe spacing of 0.268 nm is consistent with the unique separation (0.266 nm) between (120) planes in bulk acanthite Ag_2_S crystallites.

To determine the composition of the nanoparticles, the corresponding energy dispersive x-ray (EDX) spectrum of the Ag_2_S(4)/ZnO/TNTs was carried out in the HR-TEM as seen in Figure 3c. The characteristics peaks in the spectrum are associated with Ag, Ti, O, Zn, and S. The quantitative analysis reveals the atomic ratio of Ag and S is close to 2:1, indicating the deposited materials are possible Ag_2_S.

In order to determine the structure of the Ag_2_S(4)/ZnO/TNTs, the crystalline phases of the Ag_2_S(4)/ZnO/TNTs and the corresponding TNTs were characterized by XRD, as shown in Figure 3d. The XRD pattern shows peaks corresponding to TiO_2 _(anatase), ZnO (hexagon), and Ag_2_S (acanthite). The observed peaks indicate high crystallinities in the TNTs, ZnO, and Ag_2_S nanoparticles, consistent with the SEM results shown in Figure 2. The results further confirm that the obtained films are composed of TiO_2_, ZnO, and Ag_2_S.

### Optical and photoelectrochemistry properties of Ag_2_S QD-sensitized TNT electrodes in the presence of ZnO layers

**Figure 4:**
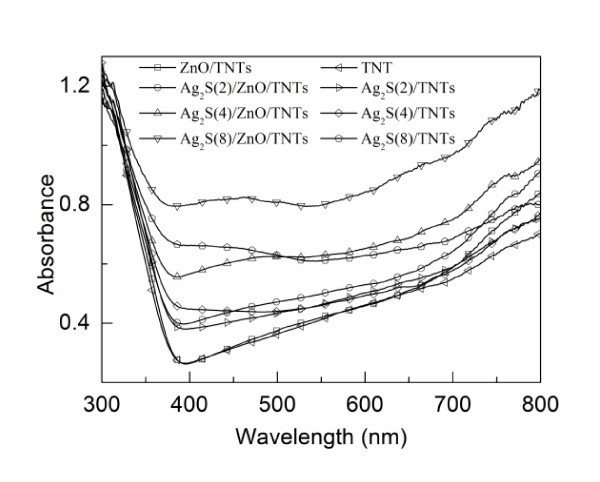
**UV-vis absorption spectrum of the plain TNT, ZnO/TNT, Ag_2_S(*n*)/TNT, and Ag_2_S(*n*)/ZnO/TNT films**. *n *= 2, 4 and 8.

Figure 4 shows optical absorption of annealed TNTs, ZnO/TNTs, and Ag_2_S(*n*)/ZnO/TNTs (*n *= 2, 4, and 8). It can be seen from Figure 4 that both plain TNTs and ZnO/TNTs absorb mainly UV light with wavelengths smaller than 400 nm. However, for the ZnO/TNT film, the absorbance of the spectra slightly increases compared to that for plain TNTs, suggesting the formation of ZnO thin film on TNTs. This result is similar to that for ZnO-coated TiO_2 _films in DSSCs [29], which can be attributed to the absorption of the ZnO layers coated on TNTs. After Ag_2_S deposition, the absorbance of the Ag_2_S(*n*)/ZnO/TNT films increases with the cycles of Ag_2_S chemical bath deposition process. Moreover, a significant shift of the spectral photoresponse is observed in the Ag_2_S(*n*)/ZnO/TNT films, indicating that the Ag_2_S deposits can be used to sensitize TiO_2 _nanotube arrays with respect to lower energy (longer wavelength) region of the sunlight. In addition, the absorbance increases with the increase in the cycles of Ag_2_S deposition, resulting from an increased amount of Ag_2_S nanoparticles.

**Figure 5:**
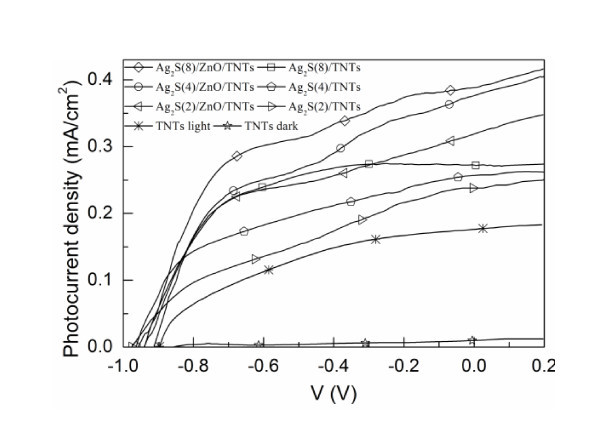
***J*-*V *characteristics of the plain TNT, Ag_2_S(*n*)/TNT, and Ag_2_S(n)/ZnO/TNT electrodes**. *n *= 2, 4, and 8.

For the performance comparison of as-prepared Ag_2_S-sensitized TNT and ZnO/TNT electrodes, the curves of photocurrent density vs. the applied potential for the Ag_2_S(*n*)/TNT and Ag_2_S(*n*)/ZnO/TNT (*n *= 2, 4, and 8) electrodes in the dark and under simulated AM 1.5 G sunlight irradiation (100 mW cm^-2^) are shown in Figure 5.

**Figure 6:**
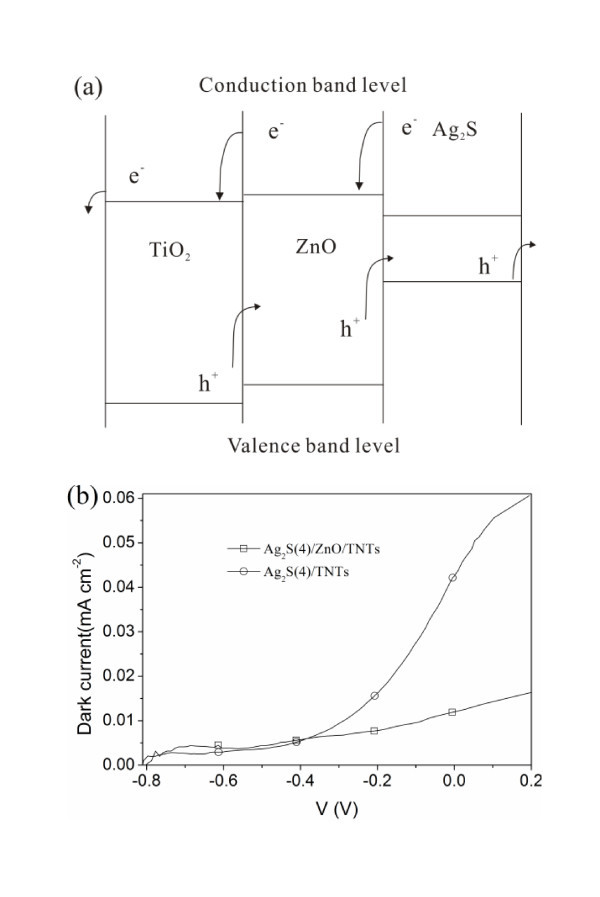
**Energy diagram and dark current**. (**a**) Energy diagram of Ag_2_S-sensitized ZnO/TNT solar cells and (**b**) the dark current of the Ag_2_S(4)/ZnO/TNT and Ag_2_S(4)/TNT electrodes.

It is clearly seen from Figure 5 that for a chemical bath deposition (CBD) cycle *n *and an applied potential, the photocurrent density of the Ag_2_S(*n*)/ZnO/TNT electrode is higher than that of the Ag_2_S(*n*)/TNTs without ZnO layer. This can be explained from the increased absorbance of the Ag_2_S(*n*)/ZnO/TNT electrode shown in Figure 4 and the energy diagram of Ag_2_S-sensitized ZnO/TNT solar cells presented in Figure 6a. Due to the formation of ZnO energy barrier layer over TNTs, the charge recombination with either oxidized Ag_2_S quantum dots or the electrolyte in the Ag_2_S-sensitized ZnO/TNT electrode is suppressed compared to the Ag_2_S-sensitized TNTs. This explanation can be supported by the dark current density-applied potential characteristics of the Ag_2_S(*n*)/ZnO/TNTs and Ag_2_S(*n*)/TNTs because the dark current represented the recombination between the electrons in the conduction band and the redox ions of the electrolyte. As an example, Figure 6b shows the curves of dark density vs. the applied potential for the Ag_2_S(4)/ZnO/TNTs and Ag_2_S(4)/TNTs. Apparently, for the Ag_2_S-sensitized TNTs with ZnO-coated layers, the dark current density decreases significantly. In addition, it is found that for both Ag_2_S-sensitized ZnO/TNT and TNT electrodes, the photocurrent density at an applied potential increases with increasing CBD cycles, which can be attributed to a higher incorporated amount of Ag_2_S that can induce a higher photocurrent density. This result is consistent with the observed UV-vis absorption spectra shown in Figure 4. Similar results have been obtained in CdS-sensitized QDSSCs [31]. Moreover, it should be noted that although the conduction band (CB) level of ZnO is slightly higher than that of TiO_2 _(Figure 6a), it seems that the electron transfer efficiency from Ag_2_S to ZnO is not much lower than that from Ag_2_S to ZnO because the photocurrent density of the Ag_2_S/ZnO/TNTs is more higher than that of the Ag_2_S/TNTs. This phenomenon can be explained as follows. According to Marcus and Gerischer's theory [32-34], the rate of electron transfer from electron donor to electron acceptor depends on the energetic overlap of electron donor and acceptor which are related to the density of states (DOS) at energy E relative to the conductor band edge, reorganization energy, and temperature. Therefore, in our case, even though The CB level of electron donor (Ag_2_S) is lower than that of electron acceptor (TiO_2 _or ZnO), the electron transfer may also happen if there is an overlap of the DOS of Ag_2_S and TiO_2 _(or ZnO), which may be the reason for the photocurrent generation in Ag_2_S-sensitized TNT electrodes. The more important thing is that for semiconductor nanoparticles, the DOS may be strongly influenced by the doped impurity [35], the size of the nanoparticles [36], and the presence of surrounding media such as liquid electrolyte (i.e., Na_2_S electrolyte in our case) [37]. This means that the DOS of semiconductor nanoparticles may distribute in a wide energy range. Recently, the calculation results [38] showed that the DOS of Ag_2_S can distribute in a wide energy range from about -14 to 5 eV, indicating that the electron can probably transfer from Ag_2_S to TiO_2 _or ZnO due to the overlap of the electric states of Ag_2_S and TiO_2 _or ZnO. Besides, considering that the difference between the CB level of TiO_2 _and that of ZnO is not so large, it may be possible that the electron transfer rate from Ag_2_S to ZnO is not much lower than that from Ag_2_S to TiO_2_. The photocurrent and photovoltage of Ag_2_S QD-sensitized TiO_2 _electrode have been experimentally found not only by us but also by others [10,25].

**Figure 7:**
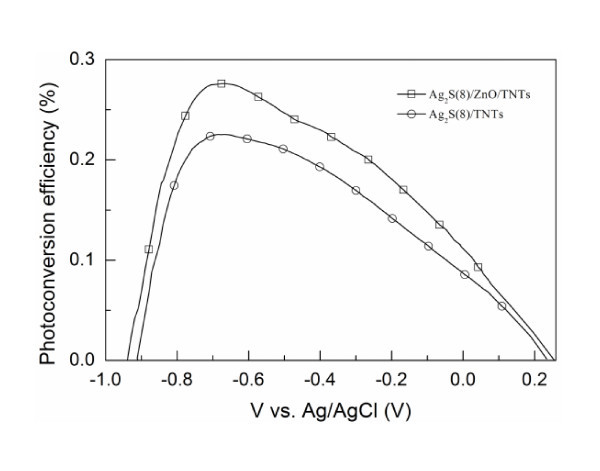
**The photoconversion efficiencies of the Ag_2_S(8)/ZnO/TNT and Ag_2_S(8)/TNT electrodes**.

Figure 7 shows the photoconversion efficiency *η *as a function of applied potential (vs. Ag/AgCl) for the Ag_2_S(8)/ZnO/TNT and Ag_2_S(8)/TNT electrodes under UV-vis light irradiation. The efficiency *η *is calculated as [39], *η *(%) = [(total power output-electric power input)/light power input] × 100 = *j*_p _[(*E*_rev _|*E*_app_|)/*I*_0_] × 100, where *j*_p _is the photocurrent density (milliamperes per square centimeter), *j*_p _× *E*_rev _is the total power output, *j*_p _× *E*_app _is the electrical power input, and *I*_0 _is the power density of incident light (milliwatts per square centimeter). *E*_rev _is the standard state-reversible potential, which is 1.23 V/NHE. The applied potential is *E*_app _= *E*_means _- *E*_aoc_, where *E*_means _is the electrode potential (vs. Ag/AgCl) of the working electrode at which photocurrent was measured under illumination and *E*_aoc _is the electrode potential (vs. Ag/AgCl) of the same working electrode under open circuit conditions, under the same illumination, and in the same electrolyte. It can be clearly seen from Figure 7 that the Ag_2_S(8)/ZnO/TNT electrode shows a higher photoconversion efficiency compared to the Ag_2_S(8)/TNT electrode with a ZnO layer for an applied potential. In particular, a maximum photoconversion efficiency of 0.28% was obtained at an applied potential of -0.67 V vs. Ag/AgCl for the Ag_2_S(8)/ZnO/TNT electrode, while it was 0.22% for the Ag_2_S(8)/TNT electrode at an applied potential of -0.67 V. The maximum photoconversion efficiency of the Ag_2_S(8)/ZnO/TNT electrode is about 1.3 times that of the Ag_2_S(8)/TNT electrode. However, it should be noted that the efficiency of the Ag_2_S-sensitized TNT electrode is worse than the value obtained from Ag_2_S QD-sensitized nanocrystalline TiO_2 _film, which was recently reported by Tubtimtae et al. [25]. The main reason may be due to the different architecture of TiO_2 _electrode. Ag_2_S QDs cannot be deposited in large numbers on the inner surface of TNTs due to the limited space in TNTs, while the number of Ag_2_S QDs deposited on the surface of nanocrystalline TiO_2 _film is almost not limited. This means that compared to the TNTs, more Ag_2_S QDs can be deposited on nanocrystalline TiO_2 _film and absorb more light leading to a higher photocurrent. Besides, in our case, we use TNT electrode and 1 M Na_2_S electrolyte. However, Tubtimtae et al. used nanocrystalline TiO_2 _film and a polysulfide electrolyte consisted of 0.5 M Na_2_S, 2 M S, 0.2 M KCl, and 0.5 M NaOH in methanol/water. Clearly, the electrolyte will affect the performance of the devices. Moreover, the photocurrent measurements are performed under different conditions. A three-electrode configuration was employed in our experiments. However, a two-electrode configuration was used in the experiments of Tubtimtae et al. In addition, our results show that the efficiency obtained from Ag_2_S-sensitized TNTs is also lower than that of CdS-sensitized TiO_2 _electrode [31]. The main reason for this may be that the CB level of Ag_2_S is lower than that of TiO_2 _as shown in Figure 6a[40], but the CB level of CdS is higher than that of TiO_2_. Therefore, the electron transfer is more efficient in CdS/TNT solar cells. The comparison of our current experiments with those by Tubtimtae et al. indicates that there is still much scope for improving the performance of the Ag_2_S-sensitied ZnO/TNT electrode. Nevertheless, our results show that the ZnO layer leads to an increased *η*.

## Conclusions

In conclusion, Ag_2_S quantum dot-sensitized TiO_2 _nanotube array photoelectrodes were successfully achieved using a simple sequential chemical bath deposition (CBD) method. In order to improve the efficiencies of as-prepared Ag_2_S quantum dot-sensitized solar cells, the Ag_2_S quantum dot-sensitized ZnO/TNT electrodes were prepared by the interposition of a ZnO energy barrier between the TNTs and Ag_2_S quantum dots. The ZnO thin layers were formed using wet-chemical process. The formed ZnO energy barrier layers over TNTs significantly increase the power conversion efficiencies of the Ag_2_S(*n*)/ZnO/TNTs due to a reduced recombination.

## Competing interests

The authors declare that they have no competing interests.

## Authors' contributions

CC carried out the experiments, participated in the sequence alignment and drafted the  manuscript. YX participated in the design of the study and performed the statistical analysis GA and SHY participated in the device preparation. SOC conceived of the study, and participated in its design and coordination. All authors read and approved the final manuscript.

## References

[B1] Grätzel M Dye-sensitized solid-state heterojunction solar cells MRS Bull 2005 30 23 27 10.1557/mrs2005.4

[B2] Wei D Dye sensitized solar cells Int J Mol Sci 2010 11 1103 1113 10.3390/ijms11031103 20480003 PMC2869240

[B3] Fan SH Wang KZ Recent advances on molecular design of ruthenium (II) sensitizers in dye-sensitized solar cells Chinese J Inorg Chem 2008 24 1206 1212

[B4] Grätzel M Dye-sensitized solar cells J Photoch Photobio C 2003 4 145 153 10.1016/S1389-5567(03)00026-1

[B5] Gao F Wang Y Shi D Zhang J Wang MK Jing XY Humphry-Baker R Wang P Zakeeruddin SM Grätzel M Enhance the optical absorptivity of nanocrystalline TiO_2 _film with high molar extinction coefficient ruthenium sensitizers for high performance dye-sensitized solar cells J Am Chem Soc 2008 130 10720 10728 10.1021/ja801942j 18642907

[B6] Vogel R Pohl K Weller H Sensitization of highly porous, polycrystalline TiO_2 _electrodes by quantum sized CdS Chem Phys Lett 1990 174 241 246 10.1016/0009-2614(90)85339-E

[B7] Niitsoo O Sarkar SK Pejoux C Ruhle S Cahen D Hodes G Chemical bath deposited CdS/CdSe-sensitized porous TiO_2 _solar cells J Photochem Photobiol A: Chem 2006 181 306 313 10.1016/j.jphotochem.2005.12.012

[B8] Diguna LJ Shen Q Kobayashi J Toyoda T High efficiency of CdSe quantum-dot-sensitized TiO_2 _inverse opal solar cells Appl Phys Lett 2007 91 023116 10.1063/1.2757130

[B9] Lόpez-Luke T Wolcott A Xu LP Chen SW Wcn ZH Li JH De La Rosa E Zhang JZ Nitrogen-doped and CdSe quantum-dot-sensitized nanocrystalline TiO_2 _films for solar energy conversion applications J Phys Chem C 2008 112 1282 1292 10.1021/jp077345p

[B10] Vogel R Hoyer P Weller H Quantum-sized PbS, CdS, Ag2S, Sb2S3, and Bi2S3 particles as sensitizers for various nanoporous wide-bandgap semiconductors J Phys Chem 1994 98 3183 3188 10.1021/j100063a022

[B11] Lee H Leventis HC Moon SJ Chen P Ito S Haque SA Torres T Nuesch F Geiger T Zakeeruddin SM Grätzel M Nazeeruddin MK PbS and CdS quantum dot-sensitized solid-state solar cells: "Old Concepts, New Results" Adv Funct Mater 2009 19 2735 2742 10.1002/adfm.200900081

[B12] Yu PR Zhu K Norman AG Ferrere S Frank AJ Nozik AJ Nanocrystalline TiO_2 _solar cells sensitized with InAs quantum dots J Phys Chem B 2006 110 25451 25454 10.1021/jp064817b 17165992

[B13] Zaban A Micic OI Gregg BA Nozik AJ Photosensitization of nanoporous TiO_2 _electrodes with InP quantum dots Langmuir 1998 14 3153 3156 10.1021/la9713863

[B14] Nozik AJ Quantum dot solar cells Physica E: Low-Dimensional Systems & Nanostructures 2002 14 115 120 10.1016/S1386-9477(02)00374-0

[B15] Roy P Kim D Lee K Spiecker E Schmuki P TiO_2 _nanotubes and their application in dye-sensitized solar cells Nanoscale 2010 2 45 59 10.1039/b9nr00131j 20648363

[B16] Xu CK Shin PH Cao LL Wu JM Gao D Ordered TiO_2 _nanotube arrays on transparent conductive oxide for dye-sensitized solar cells Chem Mater 2010 22 143 148 10.1021/cm9027513

[B17] Uchida S Chiba R Tomiha M Masaki N Shirai M Application of titania nanotubes to a dye-sensitized solar cells Electrochemistry 2002 70 418 420

[B18] Xie Y Heo SH Kim YN Yoo SH Cho SO Synthesis and visible-light-induced catalytic activity of Ag_2_S-coupled TiO_2 _nanoparticles and nanowires Nanotechnology 2010 21 015703 10.1088/0957-4484/21/1/015703 19946150

[B19] Neves MC Nogueira JMF Trindade T Mendonca MH Pereira MI Monteiro OC Organic dyes with a novel anchoring group for dye-sensitized solar cell applications J Photochem Photobiol A 2009 204 168 173 10.1016/j.jphotochem.2009.03.014

[B20] Kryukov AI Stroyuk AL Zińchuk NN Korzhak AV Kuchmii SY Optical and catalytic properties of Ag_2_S nanoparticles J Mol Catal A Chem 2004 221 209 221 10.1016/j.molcata.2004.07.009

[B21] Morales-Masis M van der Molen SJ Fu WT Hesselberth MB van Ruitenbeek JM Conductance switching in Ag_2_S devices fabricated by in situ sulfurization Nanotechnology 2009 20 095710 10.1088/0957-4484/20/9/095710 19417506

[B22] Reid M Punch J Ryan C Franey J Derkits GE Reents WD Garfias LF The corrosion of electronic resistors IEEE Tran Components and Packaging Technologies 2007 30 666 672

[B23] Wang HL Qi LM Controlled synthesis of Ag_2_S, Ag_2_Se, and Ag nanofibers by using a general sacrificial template and their application in electronic device fabrication Adv Funct Mater 2008 18 1249 1256 10.1002/adfm.200700953

[B24] Kitova S Eneva J Panov A Haefke H Infrared photography based on vapor-deposited silver sulfide thin films J Imaging Sci Technol 1994 38 484 488

[B25] Tubtimtae A Wu K Tung H Lee M Wang GJ Ag_2_S quantum dot-sensitized solar cells Electrochem Commun 2010 12 1158 1160 10.1016/j.elecom.2010.06.006

[B26] Chen C Xie Y Ali G Yoo SH Cho SO Improved conversion efficiency of CdS quantum dots-sensitized TiO_2 _nanotube array using ZnO energy barrier layer Nanotechnology 2011 22 015202 10.1088/0957-4484/22/1/015202 21135453

[B27] Lee W Kang SH Kim JY Kolekar GB Sung YE Han SH TiO_2 _nanotubes with a ZnO thin energy barrier for improved current efficiency of CdSe quantum-dot-sensitized solar cells Nanotechnology 2009 20 335706 10.1088/0957-4484/20/33/335706 19636095

[B28] Paulose M Shankar K Yoriya S Prakasam HE Varghese OK Mor GK Latempa TA Fitzgerald A Grimes CA Anodic growth of highly ordered TiO_2 _nanotube arrays to 134 μm in length J Phys Chem B 2006 110 16179 16184 10.1021/jp064020k 16913737

[B29] Roh SJ Mane RS Min SK Lee WJ Lokhande CD Han SH Achievement of 4.51% conversion efficiency using ZnO recombination barrier layer in TiO_2 _based dye-sensitized solar cells Appl Phys Lett 2006 89 253512 10.1063/1.2410240

[B30] Sun WT Yu Y Pan HY Gao XF Chen Q Peng LM CdS quantum dots sensitized TiO_2 _nanotube-array photoelectrodes J Am Chem Soc 2008 130 1124 1125 10.1021/ja0777741 18183979

[B31] Chi CF Lee YL Weng HS A CdS-modified TiO_2 _nanocrystalline photoanode for efficient hydrogen generation by visible light Nanotechnology 2008 19 125704 10.1088/0957-4484/19/12/125704 21817745

[B32] Gerischer H Charge transfer processes at semiconductor-electrolyte interfaces in connection with problems of catalysis Surf Sci 1969 18 97 122 10.1016/0039-6028(69)90269-6

[B33] Marcus RA On the theory of oxidation-reduction reactions involving electron transfer J Chem Phys 1956 24 966 978 10.1063/1.1742723

[B34] Marcus RA Chemical and electrochemical electron-transfer theory Ann Rev Phys Chem 1964 15 155 196 10.1146/annurev.pc.15.100164.001103

[B35] Feng Y Badaeva E Gamelin DR Li XS Excited-state double exchange in manganese-doped ZnO quantum dots: a time-dependent density-functional study J Phys Chem Lett 2010 1 1927 1931 10.1021/jz100402q

[B36] Lei Y Liu H Xiao W First principles study of the size effect of TiO_2 _anatase nanoparticles in dye-sensitized solar cell Modelling Simul Mater Sci Eng 2010 18 025004 10.1088/0965-0393/18/2/025004

[B37] Abayev H Zaban A Kytin VG Danilin AA Garcia-Belmonte G Bisquert J Properties of the electronic density of states in TiO_2 _nanoparticles surrounded with aqueous electrolyte J Solid State Electronchem 2007 11 647 653 10.1007/s10008-006-0220-1

[B38] Sun S Xia D An abinitio calculation study on the super ionic conductors α-AgI and Ag_2_X (X = S, Se) with BCC structure Solid State Ionics 2008 179 2330 2334 10.1016/j.ssi.2008.09.028

[B39] Khan SUM Shahry MA Ingler WBJ Efficient photochemical water splitting by a chemically modified n-TiO_2_ Science 2002 297 2243 2245 10.1126/science.1075035 12351783

[B40] Xu Y Schoonen MMA The absolute energy positions of conduction and valence bands of selected semiconducting minerals American Mineralogist 2000 85 543 556

